# Interactions between ethylene, gibberellins, and brassinosteroids in the development of rhizobial and mycorrhizal symbioses of pea

**DOI:** 10.1093/jxb/erw047

**Published:** 2016-02-17

**Authors:** Eloise Foo, Erin L. McAdam, James L. Weller, James B. Reid

**Affiliations:** ^1^School of Biological Sciences, University of Tasmania, Private Bag 55, Hobart, Tasmania 7001, Australia

**Keywords:** Arbuscular mycorrhizae, brassinosteroids, *ein2*, ethylene insensitivity, gibberellins, hormone mutants, nodulation, peas, root growth.

## Abstract

The regulation of arbuscular mycorrhizal development and nodulation involves complex interactions between the plant and its microbial symbionts. In this study, we use the recently identified ethylene-insensitive *ein2* mutant in pea (*Pisum sativum* L.) to explore the role of ethylene in the development of these symbioses. We show that ethylene acts as a strong negative regulator of nodulation, confirming reports in other legumes. Minor changes in gibberellin_1_ and indole-3-acetic acid levels in *ein2* roots appear insufficient to explain the differences in nodulation. Double mutants produced by crosses between *ein2* and the severely gibberellin-deficient *na* and brassinosteroid-deficient *lk* mutants showed increased nodule numbers and reduced nodule spacing compared with the *na* and *lk* single mutants, but nodule numbers and spacing were typical of *ein2* plants, suggesting that the reduced number of nodules in *na* and *lk* plants is largely due to the elevated ethylene levels previously reported in these mutants. We show that ethylene can also negatively regulate mycorrhizae development when ethylene levels are elevated above basal levels, consistent with a role for ethylene in reducing symbiotic development under stressful conditions. In contrast to the hormone interactions in nodulation, *ein2* does not override the effect of *lk* or *na* on the development of arbuscular mycorrhizae, suggesting that brassinosteroids and gibberellins influence this process largely independently of ethylene.

## Highlight

An ethylene-insensitive mutant of pea with enhanced nodulation and arbuscular mycorrhizal development showed that gibberellin and brassinosteroid deficiency affect nodule number via ethylene levels.

## Introduction

Symbioses between plants and microorganisms are common and are important for the acquisition of at least two key macronutrients, nitrogen and phosphorus (Ferguson *et al.*, 2010; Foo *et al.*, 2013b; Gu *et al.*, 2011). Phosphorus acquisition is enhanced by an arbuscular mycorrhizal symbiosis with fungi of the phylum Glomeromycota, a symbiosis estimated to be formed by more than 80% of land plants (Smith and Read, 2008). The symbiosis between plants and rhizobial bacteria is less common but is important for nitrogen acquisition, especially in legumes (Ferguson *et al.*, 2010). Abiotic stresses such as drought, salinity, and waterlogging influence these important symbioses and subsequent nutrient uptake, but our understanding of the specific mechanisms through which this occurs are still emerging (Belimov *et al.*, 2009; Capoen *et al.*, 2010; Larrainzar *et al.*, 2014; Singleton and Bohlool, 1984). One possibility is that these stresses may modify pathways for hormone synthesis or signalling, including stress hormones such as ethylene (Alonso *et al.*, 1999; Jones *et al.*, 2015) and growth-promoting hormones such as gibberellins and brassinosteroids (Ferguson *et al.*, 2005, 2011; Foo *et al.*, 2013a). Indeed, one recent report suggests the involvement of ethylene in the regulation of nitrogen fixation by drought (Larrainzar *et al.*, 2014). Ethylene is known to be involved in the regulation of many other aspects of plant development (Davies, 2010). Most detailed work on ethylene has been conducted in Arabidopsis, a species that does not form arbuscules when infected with mycorrhizal fungi (Veiga *et al.*, 2013) or rhizobial symbioses. The other prominent model species in ethylene research, especially in relation to fruit ripening, is tomato; while tomato forms arbuscular mycorrhizal symbioses, it does not form nodules, and is therefore not suitable for studying the effects of plant hormones on the two symbioses in the same system.

Recently, an ethylene signalling mutant, *Psein2*, was identified in pea (*Pisum sativum* L.) (Weller *et al.*, 2015), opening up the prospect of exploring the role of ethylene signalling in these symbioses in an important agricultural crop. *EIN2* encodes an N-RAMP metal-transporter-like protein and is a single-copy gene in temperate legumes such as pea and *Medicago truncatula*, unlike *Lotus japonicus* and the tropical legumes *Glycine max* and *Phaseolus vulgaris*, in which two copies occur (Miyata *et al.*, 2013; Weller *et al.*, 2015). Based on evidence from *ein2* in Arabidopsis, and the single-copy nature of *PsEIN2* (Lin *et al.*, 2009; Merchante *et al.*, 2013; Weller *et al.*, 2015), it is likely that all ethylene signalling in pea occurs through EIN2. Ethylene binding results in inactivation of the ethylene receptor CTR1, which in turn dephosphorylates EIN2 and enables its proteolytic cleavage. Release of the EIN2 C-terminal fragment allows it to enter the nucleus, where it inhibits degradation of the EIN3/EIL1 transcription factors and promotes the expression of ethylene-responsive genes (Qiao *et al.*, 2012). In the shoots of pea the product of this gene has been shown to regulate petal senescence and the response of plants to low-intensity red and blue light (Weller *et al.*, 2015), but its effects in pea on root development and symbioses with microorganisms have not been examined. Previous application studies by Lee and LaRue (1992) suggested that ethylene inhibits nodulation in peas. In *M. truncatula*, a closely related temperate legume that also forms indeterminate nodules with a persistent meristem, the *EIN2* homologue negatively regulates nodulation (Penmetsa *et al.*, 2008). The relationship between ethylene and nodulation appears more complex in legumes that form determinate nodules (Chan *et al.* 2013; Hunter, 1993: Miyata *et al.*, 2013; Schmidt *et al.*, 1999). While findings support a general negative role for ethylene, the control has recently been shown to be complex, with positive effects of ethylene early in rhizobial symbiosis (Larrainzar *et al.*, 2015). Consequently, the effect of ethylene sensitivity on nodulation still merits clarification in a wider range of legume species.

Two growth-promoting hormones, gibberellins and brassinosteroids, have been shown to increase nodule number in legumes at physiological levels (Ferguson *et al.*, 2005; Lievens *et al.*, 2005), although for gibberellin an optimum level is reached, above which a decrease in nodulation occurs (Ferguson *et al.*, 2005). These results come mainly from studies in pea, which used hormone-deficient mutants that display reduced nodulation (Ferguson *et al.*, 2005, 2011). The mutants used were *na*, which is defective in *ent*-kaurenoic acid oxidase, causing reduced gibberellin levels (Davidson *et al.*, 2003), and *lk*, which expresses a truncated steroid 5α-reductase enzyme that results in reduced brassinosteroid levels (Nomura *et al.*, 2004). These severely hormone-deficient mutants have also been shown to produce elevated levels of ethylene (Ferguson *et al.*, 2011; Ross and Reid, 1986). The elevated ethylene levels raise the possibility that at least some of the developmental changes in the mutants resulting from the reduced gibberellin and brassinosteroid levels may occur via effects on ethylene. Indeed, the shoot phenotype of the brassinosteroid-deficient *lk* mutant has some ethylene-related characteristics that can be partially reversed by treatment with the ethylene synthesis inhibitor amino-ethoxyvinyl glycine (Ferguson *et al.*, 2011; Ross and Reid, 1986). In addition, the decrease in nodulation seen in gibberellin-deficient *na* mutants can be reversed by treatment with amino-ethoxyvinyl glycine (Ferguson *et al.*, 2011), and recently it has been shown that the gibberellin biosynthetic pathway is regulated by ethylene during the development of the rhizobial symbiosis (Larrainzar *et al.*, 2015). Further studies are required to clarify the role of such interactions between ethylene, brassinosteroids, and gibberellins in the control of nodulation.

The results from studies using ethylene mutants to examine arbuscular mycorrhizae formation are inconclusive. Ethylene mutants have been examined in both *M. truncatula* and tomato. However, the reports are difficult to interpret, with the ethylene-insensitive mutant in *M. truncatula* reported to have increased mycorrhizae, and both ethylene-insensitive and ethylene-overproducing tomato plants reported to exhibit a small elevation, reduction, or no change in mycorrhizal colonization (Chan *et al.*, 2013; Riedel *et al.*, 2008; Torres de Los Santos *et al.*, 2011; Zsögön *et al.*, 2008). An inhibitory role for ethylene in mycorrhizal colonization was also suggested by enhanced colonization of mutants disrupted in the *RIN* gene, which encodes a MADS-box transcription factor that blocks ripening, including climacteric ethylene production (Torres de Los Santos *et al.*, 2011; Vrebalov *et al.*, 2002). Application studies in pea suggest that ethylene inhibits arbuscular mycorrhizal formation (Geil *et al.*, 2001) and the consensus view based on both mutant and application studies appears to be that ethylene is inhibitory for mycorrhizal formation (Geil *et al.*, 2001; Penmetsa *et al.*, 2008; Vela *et al.*, 2007). However, the role of ethylene in arbuscular mycorrhizae formation requires clarification and deserves further exploration, as ethylene signalling may be an important mechanism for plants to limit fungal colonization under stress.

To date, there has been little examination of how other hormones implicated in mycorrhizal development may interact with ethylene in the control of mycorrhizal development. Recent reports suggest a positive role for brassinosteroids in mycorrhizal development in both tomato and rice (Bitterlich *et al.*, 2014a, b). Given, as outlined above, that severe brassinosteroid deficiency causes elevated ethylene production in pea, it is important to know whether this brassinosteroid/ethylene interaction may influence mycorrhizal development.

In this paper we use the recently identified *ein2* mutant of pea (Weller *et al.*, 2015) to explore the role of the hormone ethylene on nodule and arbuscular mycorrhizal development. The *ein2* mutation eliminates the C-terminal domain of the encoded protein (Weller *et al.*, 2015), which is essential for its function in Arabidopsis (Wen *et al.*, 2012). We show that the resultant ethylene insensitivity leads to a greater number of nodules, and also to enhanced mycorrhizal colonization under conditions where ethylene levels are elevated. The *ein2* mutant does not appear to affect these symbioses by markedly altering the level of other hormones such as gibberellin_1_ (GA_1_), indole-3-acetic acid (IAA), and abscisic acid (ABA). We also generated double mutants to test the interactions between gibberellin deficiency and brassinosteroid deficiency and ethylene insensitivity on nodule number and mycorrhizal development. We show that the reduction in nodule number in gibberellin- and brassinosteroid-deficient plants is probably due to the elevated production of ethylene by these mutants. In contrast, ethylene is likely to act independently of gibberellin and brassinosteroids to influence interactions with arbuscular mycorrhizal fungi.

## Materials and methods

### Plant material and growth conditions

**Fig. 1.**
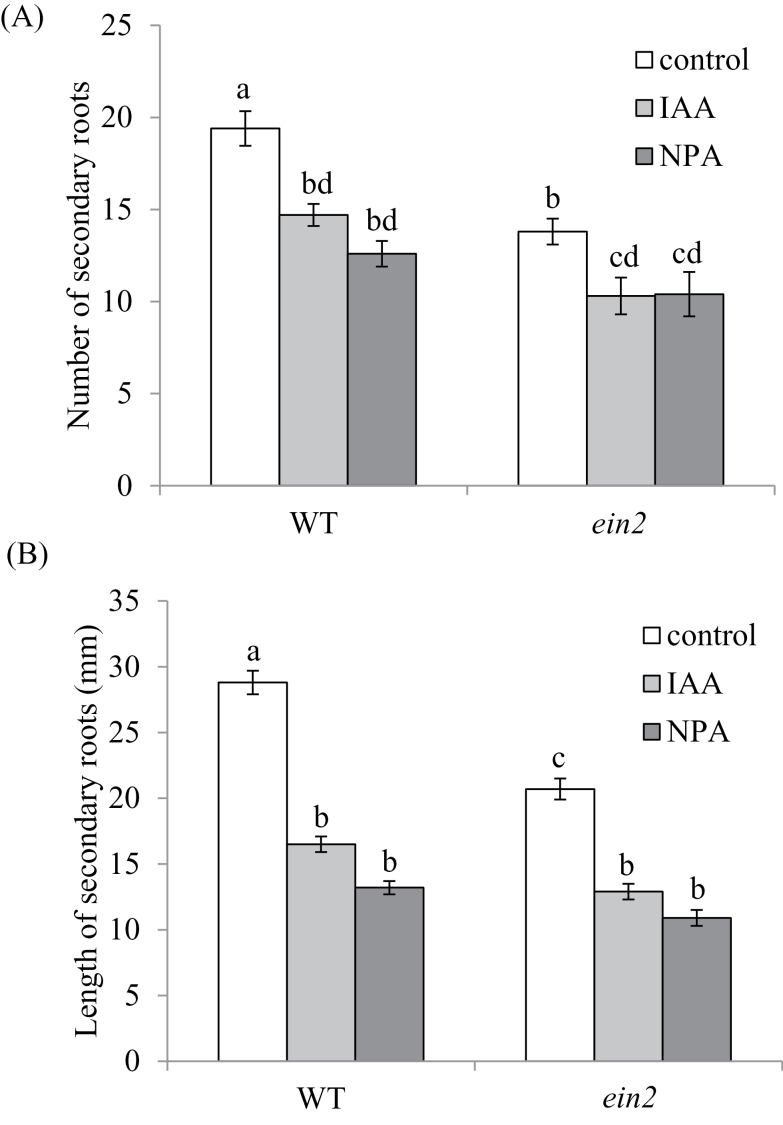
Response of roots to IAA and NPA. (A) Number and (B) average length of secondary roots of 7-day-old wild-type pea (cv. Torsdag) and *ein2* plants treated with either 1mM IAA or 50mM NPA, or left untreated. Mean±SE (*n*=9). Within each panel, values with different letters above the bars are significantly different at *P*<0.05.

The AF145 mutant line carrying the *ein2* mutation was generated by ethyl methanesulfonate mutagenesis of cv. Torsdag (Weller *et al.*, 2015) and selected after back-crossing to Torsdag three times. Crosses to produce the double mutants *ein2 na* and *ein2 lk* involved AF145 crossed to lines carrying either the *na-1* or *lk* mutant alleles that had been previously back-crossed to Torsdag six and three times, respectively. All plants were grown in 140mm pots in a 1:1 mixture of vermiculite and dolerite chips topped with 4cm of pure vermiculite in a temperature-limited glasshouse under a natural photoperiod extended to 18h. The exceptions were for plants in Fig. 1, which were grown in tubes as described by Weston *et al.* (2009) on 1.3% agar containing the appropriate level of either IAA (Sigma-Aldrich; St Louis, MO, USA) or 1-N-naphthylphthalamic acid (NPA) (Sigma-Aldrich), and for those grown for ethylene analysis as described below. For root architecture measurements, plants were grown for 14 d and root parameters were recorded, including the total number of secondary roots, the average length of the top 10 secondary roots, and the number of tertiary roots borne on these roots. Plants in the experiment receiving ethephon had either 10 μg of ethephon in 10 μl of ethanol or just 10 μl of ethanol applied every 2 weeks after planting to the uppermost expanded leaflet. This application method was used in order to achieve a minimal dose that did not markedly affect root or shoot development and to avoid any direct effect of ethephon on mycorrhizal fungi in the soil.

### Symbiosis experiments

Nodulation experiments were carried out as described in Foo and Davies (2011). Briefly, plants were grown two per pot, inoculated at day 7 with a 3-day-old culture of *Rhizobium leguminosarum* bv *viciae* (RLV248) grown in yeast-mannitol broth, and received modified Long Ashton solution containing 5mM NaH_2_PO_4_ and no nitrogen weekly. Plants were scored for phenotypic characters at the ages indicated. At the time of scoring, the total number of nodules on a root system was counted. Nodule spacing data were obtained by placing secondary roots on a grid and selecting nodules that intersected the grid, as described by Foo *et al.* (2014). Root, shoot, and nodule dry weights were obtained and nodule number expressed as the number of nodules per g root dry weight to account for small differences in root size (Foo *et al.*, 2014).

Mycorrhizae experiments were conducted as outlined in Foo *et al.* (2013a) with plants grown two per pot in vermiculite and dolerite chips containing a mixture of *Rhizophagus irregularis* spores (approximately 8000 spores per pot; Premier Tech Pty Ltd, Quebec, Canada) and propagules obtained from leek or spring onion *R. irregularis* pot cultures (1:4 by volume of inoculum to vermiculite and dolerite chips). Plants received modified Long Ashton solution containing 10mM KNO_3_ and 0.05mM NaH_2_PO_4_ weekly. Plants were removed from the soil and the whole root system was cut into 1cm segments and mixed. A subset of roots was cleared using boiling 5% KOH and stained using the ink and vinegar method (Vierheilig *et al.*, 1998). Mycorrhizal colonization of roots was scored according to McGonigle *et al.* (1990), where 150 intersects were observed from 25 root segments per plant and scored for the presence of arbuscules, vesicles, and/or intraradical hyphae. Root colonization was calculated from the percentage of intersects that contained any internal fungal structure (arbuscule, hyphae, or vesicle), and arbuscule frequency was calculated from the percentage of intersects that contained arbuscules. Some variation occurred between experiments due to seasonal affects. For experiments with *na* single mutants and *na ein2* double mutants, where some mutant lines contained an increased number of cortical cell layers compared with wild-type lines, the colonization rate for all plants was expressed relative to the number of cell layers, as described in Foo *et al.*, (2013a).

### Hormone analyses

Levels of IAA and GA_1_ were determined from mature whole roots as described by Jager *et al.* (2005, 2008). Briefly, samples were homogenized and extracted into 80% (v/v) methanol and deuterated internal standards were added. Samples were passed through C_18_ Sep-Pak cartridges (Waters, Australia) and fractionated using a reverse-phase C_18_ HPLC system (Waters Associates, Milford, USA). The individual fractions containing gibberellins, IAA, or ABA were pooled separately. Gibberellin-containing samples were methylated. Gibberellin and IAA samples were then analysed by gas chromatography-mass spectrometry-selected ion monitoring using a Hewlett Packard 5890 gas chromatograph coupled to a Kratos Concept ISQ mass spectrometer controlled by a Mach 3 data system (Jager *et al.*, 2005). Pooled fractions containing ABA were partitioned against diethyl ether, resuspended in chloroform, and GC-MS-MS analysis was performed with a Varian 8400 Autosampler and a Varian 3800 GC coupled to a Varian 1200 triple quadrupole MS, as described by Jager *et al.* (2008). Endogenous hormone levels were calculated from the ratio of endogenous to standard peak areas after mass spectroscopy and normalized to per g root fresh weight.

For ethylene analysis, wild-type and *ein2* mutant plants were grown three per jar in unsealed 600ml glass jars, filled to one-third of the volume with damp vermiculite. Plants were grown in a growth cabinet under an 18h photoperiod (100 µmol quanta m^−2^ s^−1^ at the top of the jar) at 20 °C/15 °C day/night temperatures. After 11 d, when plants had two expanded leaves, each jar was sealed with a gas-tight lid fitted with a septum. After a further 30h growth in the cabinet, 400 µl samples of headspace gas were withdrawn with syringes from four replicate jars of each genotype. GC-MS was performed as described by Foo *et al.* (2006). A known concentration of acetylene gas (4 ppm in 100 µl) was mixed with each sample and a standard of mixed ethylene and acetylene (both 4 ppm in 500 µl) was analysed between each replicate so that the concentration of ethylene in the samples could be calculated. After analysis, the plants were removed from the jars and whole-plant fresh weight was determined.

### Data analysis

Statistical analyses were performed using Excel Stat Plus. Percentage data were arc-sin transformed before analysis. For pairwise comparisons *t*-tests were performed, and for all other analyses ANOVAs were performed followed by Tukey’s post-hoc test (*P*<0.05).

## Results

### Root phenotype and the response to auxin of a pea *ein2* mutant

Although the *ein2* mutant does not display a classical triple response to applied ethylene (Goeschl *et al.*, 1967; Weller *et al.*, 2015), the roots of *Psein2* mutants have only a subtle phenotype. The secondary roots are slightly shorter than those of the wild-type progenitor cv. Torsdag (*P*<0.05, Table 1) and the number of tertiary roots is reduced (*P*<0.01) when grown in potting medium, but there is no effect on the ratio of shoot to root fresh weights. There is also a reduction in secondary root numbers and lengths in some circumstances, such as when seedlings are grown in tubes on agar medium (*P*<0.01, Fig. 1). Auxin plays a central role in the regulation of root growth in many species, and several authors have suggested that it may do so in part by modulating ethylene levels or response (Clark *et al.*, 1999; Stepanova *et al.*, 2007). Application of the auxin IAA and an inhibitor of auxin transport, NPA, significantly inhibited secondary root number and length in wild-type peas (Fig. 1). A similar response was observed in the *ein2* mutant. There was no significant interaction between genotype and treatment in a two-way ANOVA. This suggests that these compounds do not act primarily in the roots by affecting EIN2-dependent ethylene signalling, in agreement with the work of Eliasson *et al.* (1989). However, Prayitno *et al.* (2006) have shown that in Medicago *Mtein2* can influence auxin transport and subsequent nodulation.

**Table 1.** The effect of ein2 on root growth

Genotype	Number of secondary roots	Average length of top 10 secondary roots (mm)	Number of tertiary roots	Shoot FW: root FW
WT	71.5±8.3	136.0±4.5	5.7±0.6	1.3±0.2
*ein2*	82.5±6.03	123.0±2.8*	2.8±0.2**	1.1±0.1

Comparison of the parental wild-type (WT) cv. Torsdag with the *ein2* mutant grown under glasshouse conditions for 14 d for the number of secondary and tertiary roots, the average length of the uppermost 10 secondary roots per plant, and the shoot fresh weight:root fresh weight ratio. Mean±SE (*n*=4–8). * *P*<0.05, ** *P*<0.01.

### Hormone levels in the *ein2* mutant

To examine whether ethylene insensitivity may influence root development by modifying the levels of other hormones known to regulate root growth, we examined bioactive GA_1_, IAA, and ABA levels in *ein2* roots. No substantial differences in the levels of these hormones (see Ross *et al.*, 2011) were observed between the wild-type plants and the *Psein2* mutant roots, even though in the cases of IAA and GA_1_ they were significant at the 5% level (Table 2). We also determined the level of ethylene produced by *ein2* plants and found that the level was significantly increased, by approximately twofold, compared with wild-type plants (Supplementary Fig. S1 at *JXB* online). This is similar to the increase seen in *Atein2* plants (Guzmán and Ecker, 1990) and is typical of the feedback mechanisms seen in hormone-insensitive mutants (e.g. Nomura *et al.*, 1999).

**Table 2.** Hormone levels in whole mature roots

Genotype	GA_1_ (ng g^–1^ FW root)	IAA (ng g^–1^ FW root)	ABA (ng g^–1^ FW root)
WT	0.11±0.004	10.78±0.2	8.1±2.8
*ein2*	0.078±0.004*	12.0±0.3*	5.8±1.2

Levels of GA_1_, IAA, and ABA in whole root systems of glasshouse-grown 18-day-old wild-type (WT) cv. Torsdag plants and *ein2* mutants. Mean±SE (*n*=3). * *P*<0.05.

### Symbiotic development in *Psein2* mutants

**Fig. 2.**
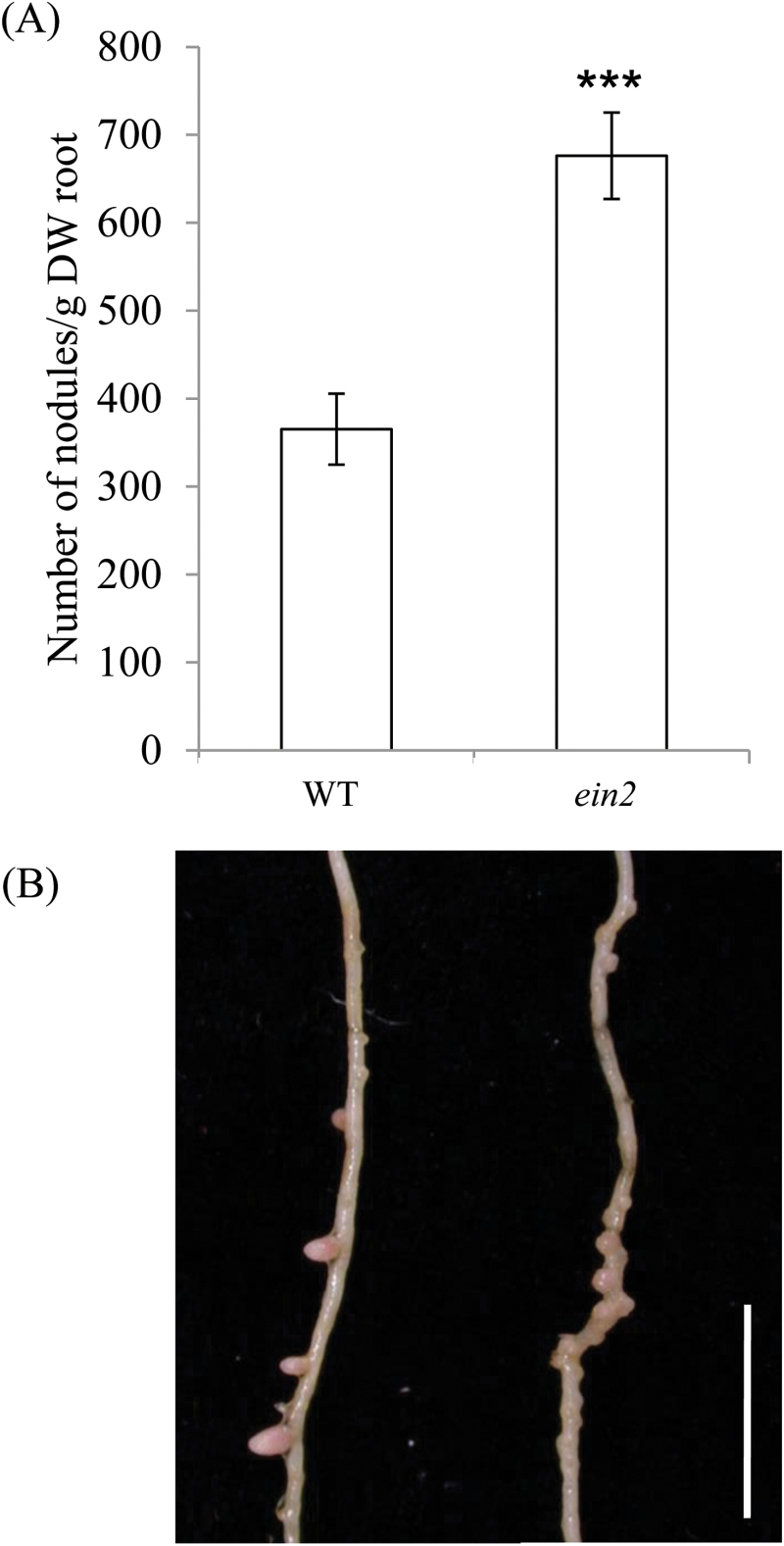
Nodulation in *ein2* and wild-type plants. (A) Number of nodules per g dry root weight. Values are mean±SE (*n*=5). ***P<0.001. (B) Photograph of nodules on a secondary root of (left) wild-type and (right) *ein2* plants (tertiary roots have been removed) grown under nitrate-free conditions for 28 days. Scale bar=1cm. (This figure is available in colour at *JXB* online.)

In pea, *Psein2* mutants exhibit a significant increase in the number of nodules formed for a given root mass compared with wild-type plants (*P*<0.001; Fig. 2). These nodules are smaller than those on wild-type plants and are more closely spaced (Fig. 2B), a characteristic of nodulation in ethylene-insensitive lines (Miyata *et al.*, 2013; Penmetsa and Cook, 1997). Our results from pea, a temperate species with indeterminate nodules, therefore support an inhibitory role of ethylene in nodule formation (e.g. Lee and LaRue, 1992, Penmetsa and Cook, 1997).

**Fig. 3.**
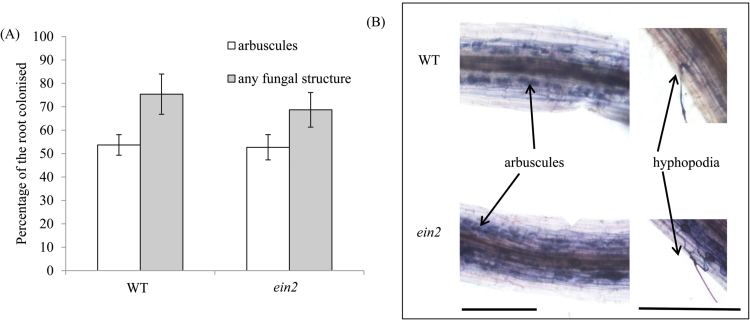
Mycorrhizal colonization of roots of wild-type and *ein2* mutants. The plants were grown for 6 weeks in pot culture with *Rhizophagus irregularis* under low phosphate (0.05mM NaH_2_PO_4_). (A) The roots were scored for the percentage of roots possessing arbuscules or any internal fungal structures (hyphae, arbuscules, and/or vesicles). Values are mean±SE (*n*=6). (B) Photomicrograph of colonized roots showing arbuscules and hyphopodia. Scale bars=100 µm. (This figure is available in colour at *JXB* online.)

As outlined previously (Foo *et al.*, 2013a), a generally negative role for ethylene in mycorrhizal symbioses has been reported. We found no significant effect of the *ein2* mutation on mycorrhizal colonization in pea when grown in relatively unstressed conditions in pots. When grown with *R. irregularis* fungi, *Psein2* roots were colonized to a similar extent to wild-type roots (Fig. 3A), including a similar percentage of the root containing arbuscules (the nutrient exchange unit of the symbiosis) (Fig. 3A), and formed normal internal fungal structures (hyphae and arbuscules; Fig. 3B). Hyphopodia, when observed, also appeared normal (Fig. 3B).

**Fig. 4.**
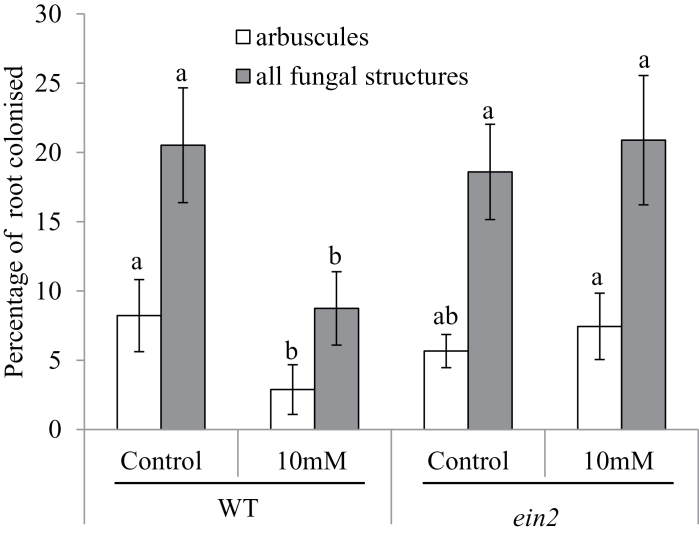
Mycorrhizal colonization of roots treated with ethephon. Wild-type and *ein*2 mutants were treated with 0 (control) or 10 μg of ethephon applied to the leaf fortnightly. Plants were grown for 6 weeks in pot culture with *Rhizophagus irregularis* under low phosphate (0.05mM NaH_2_PO_4_) and scored for the percentage of roots possessing arbuscules or any internal fungal structures (hyphae, arbuscules, and/or vesicles). Values are mean±SE (*n*=6). Within a parameter, values with different letters above the bars indicate differences at *P*<0.05.

In order to examine whether *ein2* mutants had altered mycorrhizal colonization under elevated ethylene, as may be experienced by plants under stress, we treated wild-type and *ein2* mutants with ethephon (Fig. 4), an ethylene-releasing compound (Yang, 1969). Ethephon significantly suppresses overall shoot and root growth in wild-type plants, but *ein2* plants show no significant response (Weller *et al.*, 2015; data not shown). Treatment with ethephon resulted in a significant reduction in the percentage of the root colonized with fungal structures and arbuscules in wild-type plants; this response was absent in *ein2* mutants (Fig. 4). A two-way ANOVA confirmed this interaction between genotype and ethephon treatment for the percentage of the root colonized by fungal structures (*P*<0.05), indicating that ethylene may be a negative regulator of mycorrhizal colonization in pea when ethylene levels are elevated above the baseline level.

### Gibberellin and brassinosteroid deficiency affect nodule number via ethylene

Bioactive gibberellins and brassinosteroids can act as positive regulators of nodulation in pea (Ferguson *et al.*, 2005). The gibberellin-deficient extreme dwarf *na* mutant forms a small number of incompletely developed nodules, while the brassinosteroid-deficient *lk* mutant has a reduced number of nodules, which may be larger than those on wild-type plants and appear fully functional (Ferguson *et al.*, 2005). However, both *na* and *lk* mutant plants have been shown to produce more ethylene than comparable wild-type lines, and some aspects of their phenotypes appear to at least be partly due to these elevated ethylene levels (Ferguson *et al.*, 2011; Ross and Reid, 1986). In order to examine the interactions between these hormones, the double mutants *ein2 lk* and *ein2 na* were produced and their nodulation phenotypes examined.

**Fig. 5.**
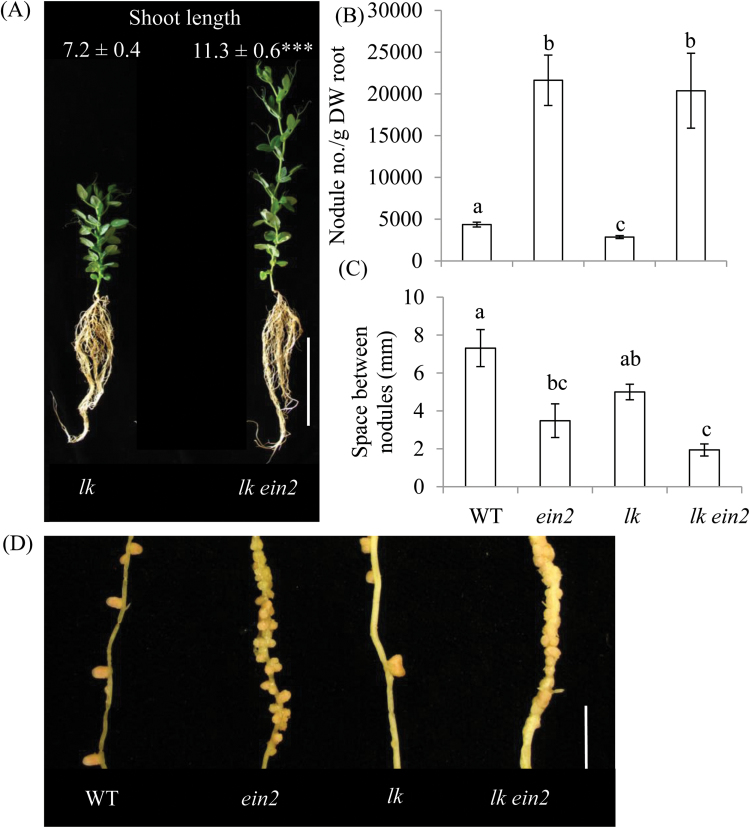
Nodulation in wild-type, *ein2*, *lk* and *lk ein2* double mutant plants. Plants were grown under nitrate-free conditions for 35 days. (A) Photograph of whole plants, including shoot height (length between nodes 1 and 9 in cm; scale bar=5cm). (B) Number of nodules per g dry weight of roots. (C) Average space between nodules. (D) Photograph of nodules on a secondary root of wild-type, *ein2, lk*, and *lk ein2* plants (tertiary roots have been removed; scale bar=1cm). For A *** *P*<0.001; for B and C, values with different letters above the bars indicate differences at *P*<0.05. Values are mean±SE (*n*=6). (This figure is available in colour at *JXB* online.)

The *ein2 lk* double mutant plants had a significantly different shoot phenotype from those of the single mutant *lk* plants. The internodes of the *ein2 lk* plants were more than 50% longer than those of the single-mutant *lk* plants (*P*<0.001, Fig. 5A). However, the plants were still much shorter than wild-type or single-mutant *ein2* plants (34% and 37% of the length respectively; data not shown). This clearly shows that while the elevated level of ethylene produced by *lk* plants (Ross and Reid, 1986) has a minor effect on shoot elongation, the major effect on elongation caused by brassinosteroid deficiency is not due to elevated ethylene levels. This is consistent with the stimulatory effect of ethylene synthesis inhibitors on the shoot growth of a brassinosteroid-deficient mutant (Ross and Reid, 1986).

As previously reported, nodule number was significantly reduced in the single *lk* mutant plants and significantly elevated in single *ein2* mutant plants compared with wild-type plants (Ferguson *et al.*, 2005; Figs. 2 and 5B, D). For *ein2*, this was also reflected in a decrease in nodule spacing (Fig. 5C). The nodules on *lk* plants were similar in weight to those on wild-type plants and pink in colour, suggesting that they were functional (Fig. 5D; data not shown). The *ein2* mutation appears to be fully epistatic to *lk* in terms of nodule number, despite the fact that *lk ein2* double-mutant shoots are very much reduced in size compared with those of wild-type and *ein2* plants. This suggests a direct effect of the ethylene insensitivity on nodule number. However, nodules on *lk ein2* double mutants were pink and appeared to be functional (Fig. 5D). This suggests that brassinosteroids may be stimulatory for nodule initiation via an effect on ethylene levels but do not affect subsequent nodule development.

**Fig. 6.**
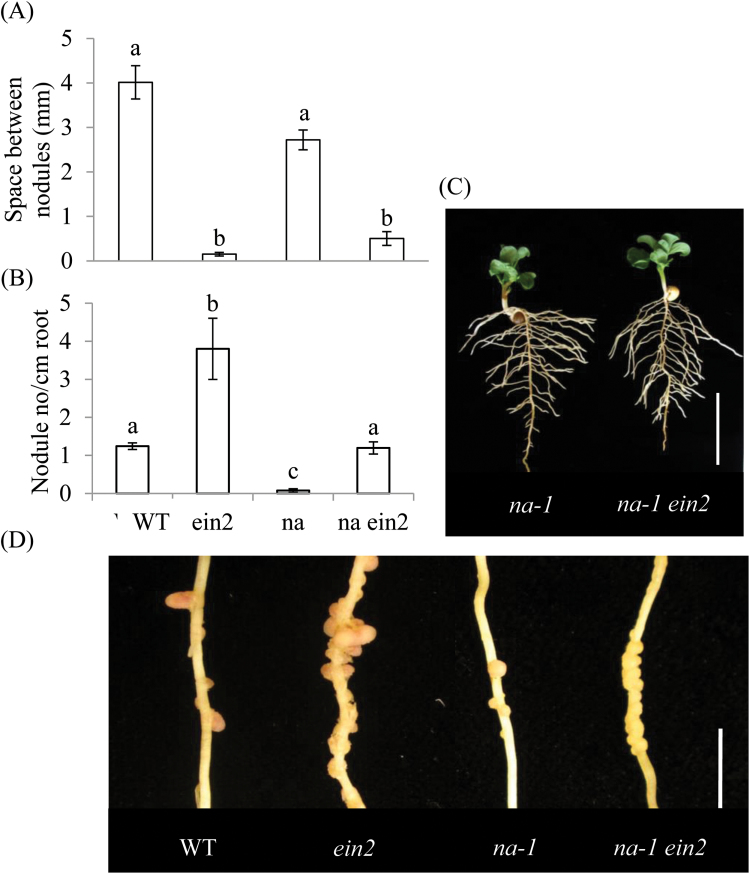
Nodulation in wild-type, *ein2*, *na*, and *na ein2* double-mutant plants. Plants were grown under nitrate-free conditions for 32 days. (A) Average space between nodules. (B) Number of nodules per cm of root. (C) Photograph of whole plants of *na* and *na ein2* (scale bar=5cm). (D) Photograph of nodules on a secondary root (tertiary roots have been removed; scale bar=1cm). Values with different letters above the bars indicate differences at *P*<0.05. Values are mean±SE (*n*=6). (This figure is available in colour at *JXB* online.)

The *ein2 na* double-mutant plants had an extremely dwarfed shoot, similar to those of the *na* plants, showing that ethylene is not involved with the manifestation of this gibberellin-deficient phenotype (Fig. 6). The roots of double-mutant plants were also still thickened and showed reduced branching compared with wild-type plants (data not shown), phenotypically similar to the root phenotype of the single-mutant *na* plants (Fig. 6C; Ferguson *et al.*, 2011). However, the *ein2 na* roots had a distinctly different nodulation phenotype from those of *na* single-mutant plants (Fig. 6A–D). The spacing between nodules was dramatically reduced in the double mutant compared with wild-type plants, almost to the same extent as in the *ein2* plants (Fig. 6A). Double-mutant plants also displayed an increased number of nodules per cm of root compared with *na* single-mutant plants (Fig 6B). The effect of *na* on nodule number is well understood (Ferguson *et al.*, 2005, 2011) and was obvious in these plants (Fig. 6). Overall, these results suggest that the elevated level of ethylene in *na* plants could be at least partly responsible for the substantial reduction in nodule number. However, the nodules produced on the *ein2 na* plants were still small and white in colour (Fig. 6D), suggesting that they were non-functional, unlike the nodules on *ein2 lk* plants. This suggests that while gibberellin levels influence nodule number at least partially through ethylene levels and subsequent EIN2-dependent ethylene signalling (similar to brassinosteroid-deficient *lk* plants), gibberellins also appear to be involved with subsequent nodule development independent of ethylene signalling.

### Lack of interaction of brassinosteroids and gibberellins with ethylene during mycorrhizal development

Recent reports suggest that brassinosteroids promote mycorrhizal development in tomato and rice (Bitterlich *et al.*, 2014a, b), while gibberellin signalling suppresses arbuscule development, including in pea (Floss *et al.*, 2013; Foo *et al.*, 2013a; Yu *et al.*, 2014). In pea, the brassinosteroid mutant *lkb* causes a partial block in the conversion of 24-methylenecholesterol to campesterol during brassinosteroid biosynthesis (Nomura *et al.*, 1999) due to a mutation in the pea homologue of *DIM* in Arabidopsis (Schultz *et al.*, 2001). This mutation does not influence arbuscular mycorrhizal development, but it is not clear whether this is due to the leaky nature of the mutation or that brassinosteroids do not influence mycorrhizal development in pea (Foo *et al.*, 2013a).

**Fig. 7.**
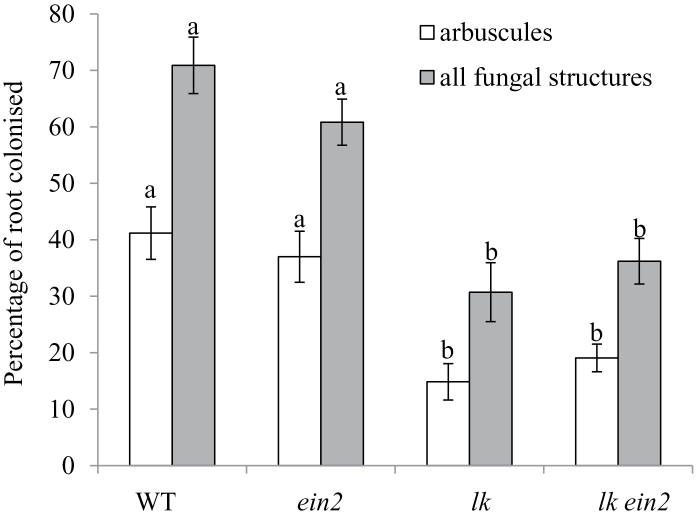
Mycorrhizal colonization of wild-type, *ein2*, *lk*, and *lk ein2* plants. Plants were grown for 6 weeks in pot culture with *Rhizophagus irregularis* under low phosphate (0.05mM NaH_2_PO_4_) and the roots were scored for the percentage of roots possessing arbuscules or any internal fungal structures (hyphae, arbuscules, and/or vesicles). Within a parameter values, with different letters above the bars indicate differences at *P*<0.01. Values are mean±SE (*n*=7–11).

To differentiate between these two alternatives, we used the more severe *lk* mutant of pea (Reid, 1986; Ross and Reid, 1989), which carries a putative null mutation in the ortholog of the steroid 5α-reductase *DET2* and shows a dramatic reduction in brassinosteroid levels (Nomura *et al.*, 2004). The results clearly show that the *lk* mutation reduces total root colonization by the fungus, including a significant decrease in the percentage of roots containing arbuscules compared with wild-type roots (Fig. 7; *P*<0.01). This is consistent with the findings in tomato and rice (Bitterlich *et al.*, 2014a, b), although the previous report in the leaky *lkb* mutant of pea (Foo *et al.*, 2013a) suggests that the reduction in brassinosteroid level must be large before an effect is observed. This supports the hypothesis that brassinosteroids do influence mycorrhizal development in pea.

As outlined in the previous section, both *na* and *lk* mutant plants have been shown to produce more ethylene than comparable wild-type lines, and this may account for at least part of their reduced nodulation phenotypes (Figs 5 and 6). We also examined whether elevated ethylene may influence the mycorrhizal phenotype of these gibberellin- and brassinosteroid-deficient lines by examining mycorrhizal development in the double mutants containing the *ein2* ethylene-insensitive mutation.

**Fig. 8.**
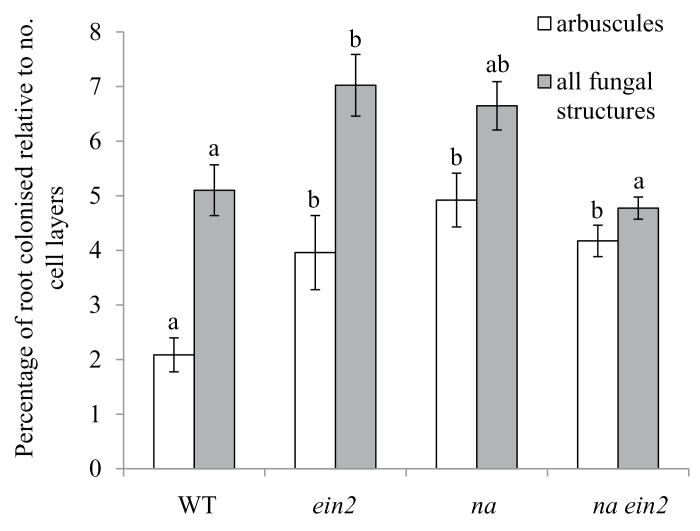
Mycorrhizal colonization of wild-type, *ein2*, *na*, and *na ein2* plants. Plants were grown for 6 weeks in pot culture with *Rhizophagus irregularis* under low phosphate (0.05mM NaH_2_PO_4_). The roots were scored for the percentage of roots possessing arbuscules or any internal fungal structures (hyphae, arbuscules, and/or vesicles), and this is expressed relative to the number of cortical cell layers to account for the increase in cortical cell layers in roots of *na* and *na ein2* mutants compared with wild-type plants and the *ein2* mutant. Within a parameter, values with different letters above the bars indicate differences at *P*<0.05. Values are mean±SE (*n*=7–11).

Interestingly, in one of these experiments we observed a small but significant increase in the percentage of the root colonized by fungi and arbuscules in *ein2* single-mutant plants compared with wild-type plants (Fig. 8; *P*<0.05). This is in contrast to previous experiments, where no effect of *ein2* was observed (Figs 3, 4, and 7). It is possible that this was due to somewhat higher ethylene production in this particular experiment, which might have caused a small suppression of mycorrhizal development in ethylene-sensitive wild-type plants, as seen for ethephon treatment in Fig. 4. However, elevated ethylene could not explain the decrease in mycorrhizal colonization observed in *lk* mutant plants, as colonization of the *lk ein2* double mutant did not differ from that of single-mutant *lk* plants (Fig. 7). Indeed, like *lk* single mutants, *lk ein2* double mutants formed significantly fewer arbuscules than wild-type plants. Likewise, the increase in arbuscule development in single-mutant *na* plants (compared with wild-type plants, *P*<0.05; Fig. 8) is similar to that observed in *na ein2* double mutants (Fig. 8). These data suggest that brassinosteroids and gibberellins each have a primary effect on mycorrhizal colonization rather than acting indirectly through altered ethylene production.

## Discussion

While there has been debate in the literature about the role of ethylene in both rhizobial and arbuscular mycorrhizal symbioses, the results with the *Psein2* mutant are clear and generally in agreement with the results seen in the Medicago *Mtein2* mutant (Penmetsa and Cook, 1997; Penmetsa *et al.*, 2008). Nodulation is dramatically increased in the *Psein2* mutant, and this closely aligns with the conclusion from mutant studies in *M. truncatula* (Larrainzar *et al.*, 2015; Penmetsa and Cook, 1997) and *L. japonicus* (Miyata *et al.*, 2013) that ethylene is a negative regulator of nodulation. Indeed, localized production of ethylene has been shown to regulate nodule positioning (Chan *et al.*, 2013). The contrary results in relation to the role of ethylene in nodulation from other legumes (e.g. soybeans; Hunter, 1993) or other reports in *L. japonicus* possibly reflect redundancy in the EIN2 pathway (Chan *et al.*, 2013; Desbrosses and Stougaard, 2011). A promotive effect of *Psein2* on arbuscular mycorrhizal colonization is seen under stressful conditions or when ethylene levels have been raised by the addition of ethephon (Fig. 4). Overall, it appears that ethylene can act as a negative regulator of arbuscular mycorrhizal colonization, as was shown by Geil *et al.* (2001). However, in contrast to its effects on nodulation, in pea ethylene levels may need to reach a threshold above the basal level to influence arbuscular mycorrhizal colonization, as might occur under stressful environmental conditions; this may explain why, in contrast to *Mtein2* mutants (Penmetsa *et al.*, 2008), *Psein2* mutants do not always display elevated mycorrhizal development.

Ethylene insensitivity, conferred by the *Psein2* mutation, has only a minor effect on root growth in pea under glasshouse conditions (Table 1), indicating that altered root development is not the cause of the altered symbiosis phenotypes of this mutant. Basal ethylene levels in wild-type peas do not appear to substantially limit root growth, and the response seen in *Psein2* plants is consistent with the effects of ethylene insensitivity in other species such as Arabidopsis (Stepanova *et al.*, 2007) and *M. truncatula* (Penmetsa and Cook, 1997). We also found no evidence that ethylene signalling through EIN2 is necessary for IAA-induced inhibition of root growth in pea plants grown *in vitro* (Fig. 1), consistent with previous reports in Arabidopsis (Stepanova *et al.* 2007).

The gibberellin-deficient *na* plants and the brassinosteroid-deficient *lk* plants both have substantially dwarfed shoot phenotypes compared with wild-type and *ein2* plants. However, dwarfism was not correlated with the symbiosis phenotypes observed in the roots, because in an *ein2* background dwarf *na* (Fig. 6) and *lk* (Fig. 5) plants had substantially increased nodule numbers (and reduced nodule spacing) compared with single-mutant *na* or *lk* parents. Furthermore, *lk* was epistatic to *ein2* in terms of the extent of mycorrhizal colonization of the roots (Fig. 7). This suggests that the effect of gibberellins and brassinosteroids on symbioses is not an indirect effect of their influence on general plant growth, but may represent a more direct developmental regulation.

While there is strong evidence that gibberellin and brassinosteroid levels influence ethylene levels in *na* and *lk* plants (Ferguson *et al.*, 2011; Ross and Reid, 1986), there is little evidence that the loss of ethylene sensitivity in *ein2* plants meaningfully alters the level of GA_1_, IAA, or ABA by a physiologically relevant amount (Table 2; see Ross *et al.*, 2011). This suggests that the observed effects of *ein2* on nodulation do not result from changes in the level of these hormones, at least at the gross whole-root level. However, the increased nodulation of double-mutant *ein2 lk* and *ein2 na* plants suggests that gibberellins and brassinosteroids influence ethylene levels, which in turn influence nodule numbers.

In contrast, there is no clear evidence that gibberellins or brassinosteroids interact with ethylene to regulate the symbiosis with arbuscular mycorrhizal fungi. Studies in several species, including pea, indicate that gibberellin signalling through DELLA proteins inhibits arbuscule development (Floss *et al.*, 2013; Foo *et al.*, 2013a; Yu *et al.*, 2014). Consistent with this, severely gibberellin-deficient *na* mutants of pea have increased arbuscule formation (Foo *et al.*, 2013a; Fig. 8). We found that double-mutant *na ein2* plants also displayed elevated arbuscule formation, indicating that elevated ethylene cannot explain the *na* mycorrhizal phenotype and that gibberellin is likely to act directly on mycorrhizal development. In contrast, the low arbuscular mycorrhizal colonization rates observed in *lk* mutants (Fig. 7) are consistent with their elevated ethylene production (Ross and Reid, 1986). However, like *lk* single mutants, *lk ein2* double mutants still displayed reduced arbuscular mycorrhizal colonization compared with wild-type plants (Fig. 7), indicating that brassinosteroid deficiency has a more direct effect on arbuscule formation, rather than via an indirect effect on ethylene signalling.

**Fig. 9.**
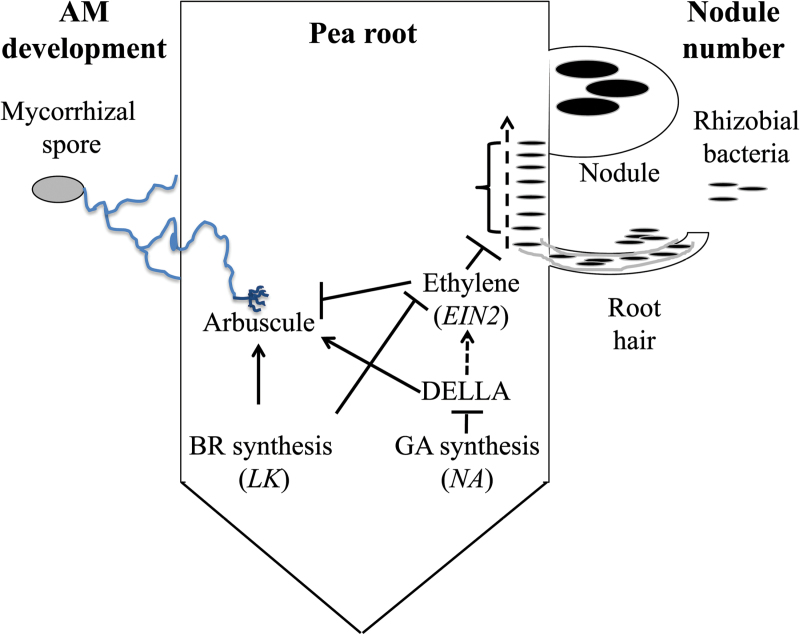
Model for the roles of gibberellin (GA), brassinosteroid (BR), and ethylene in nodule number and arbuscular mycorrhizal (AM) development in pea. Flat-ended lines indicate a negative influence, while arrows indicate a positive influence. (This figure is available in colour at *JXB* online.)

In conclusion, genetic studies using an *ein2* mutant of pea show that ethylene influences nodule number and arbuscular mycorrhizal colonization. The characterization of double mutants combining gibberellin or brassinosteroid deficiency with *ein2* suggests that a major part of the effect of these hormones on nodule number may be due to changes in ethylene levels, but this is not the case for their effects on colonization by mycorrhizal fungi. The fact that we have been able to delineate different interactions and roles for gibberellin, brassinosteroids, and ethylene in the regulation of nodule number and arbuscular mycorrhizal colonization is intriguing, and these interactions and the proposed sequence of their actions and the genes involved are outlined in Fig. 9. Although ethylene generally acts to inhibit these carbon-intensive symbioses, how elevated ethylene, such as that generated when plants are under stress, may impinge upon these processes may also be influenced by the status of other hormones. Given that both gibberellin and brassinosteroids have been implicated in protection against stress, including drought, oxidative stress, and salinity (e.g., Bajguz and Hayat, 2009; Colebrook *et al.*, 2014; Jager *et al.*, 2008; Kagale *et al.*, 2007; Kohli *et al.*, 2013), such an insight may be important for future studies examining how plants balance symbioses with plant growth under stressful conditions.

## Supplementary data

Figure S1. Endogenous ethylene level emitted by 12-day-old wild-type and *ein2* mutant pea (*Pisum sativum*) plants.

Supplementary Data
