# Supplementary material for: Interactions between ethylene, gibberellins, and brassinosteroids in the development of rhizobial and mycorrhizal symbioses of pea
Source: J Exp Bot. 2016 Feb 17;67(8):2413–24. doi: 10.1093/jxb/erw047 (PMC4809293; doi:10.1093/jxb/erw047)
Supplement: Supplementary Data [file supp_67_8_2413__index.html]

Interactions between ethylene, gibberellins, and brassinosteroids in the development of rhizobial and mycorrhizal symbioses of pea — Interactions between ethylene, gibberellins, and brassinosteroids in the development of rhizobial and mycorrhizal symbioses of pea — Supplementary Data 

# Interactions between ethylene, gibberellins, and brassinosteroids in the development of rhizobial and mycorrhizal symbioses of pea

## Supplementary Data

Data files

- supplementary\_figure\_S1.pdf - Supplementary Data
