# Supplementary material for: Interactions between ethylene, gibberellins, and brassinosteroids in the development of rhizobial and mycorrhizal symbioses of pea
Source: J Exp Bot. 2016 Feb 17;67(8):2413–24. doi: 10.1093/jxb/erw047 (PMC4809293; doi:10.1093/jxb/erw047)
Supplement: Supplementary Data [file supp_erw047_supplementary_figure_S1.pdf]

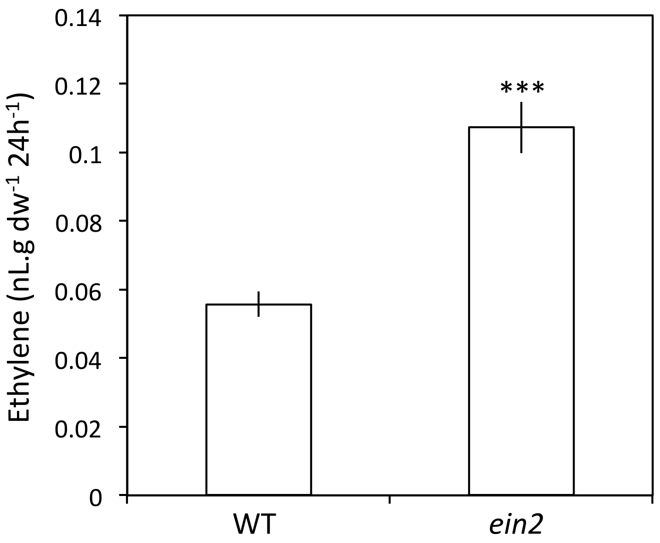

Fig. S1. Endogenous ethylene level emitted by 12-d-old wild type and *ein2* mutant pea (*Pisum sativum*) plants. Values are means ± SE (n = 4).
